# Oil supplementation improved growth and diet digestibility in goats and sheep fed fattening diet

**DOI:** 10.5713/ajas.18.0059

**Published:** 2018-07-26

**Authors:** Su Chui Len Candyrine, Mohammad Faseleh Jahromi, Mahdi Ebrahimi, Wei Li Chen, Siamak Rezaei, Yong Meng Goh, Norhani Abdullah, Juan Boo Liang

**Affiliations:** 1 Laboratory of Sustainable Animal Production and Biodiversity, Institute of Tropical Agriculture and Food Security, Universiti Putra Malaysia, Serdang 43400, Malaysia; 2 Faculty of Sustainable Agriculture, Universiti Malaysia Sabah, Sandakan Campus, Sandakan, Sabah 90509, Malaysia; 3 Agriculture Biotechnology Research Institute of Iran, East and North-East Branch, Mashhad 844, Iran; 4 Department of Veterinary Pre-Clinical Science, Faculty of Veterinary Medicine, Universiti Putra Malaysia, Serdang 43400, Malaysia; 5 Center of Emerging and Re-emerging Infectious Diseases in Animals, Faculty of Veterinary Science, Chulalongkorn University, Bangkok 10330, Thailand

**Keywords:** *Caprine*, *Ovine*, Polyunsaturated Fatty Acids, Growth Studies, Rumen Fermentation

## Abstract

**Objective:**

This study evaluated the growth, digestibility and rumen fermentation between goats and sheep fed a fattening diet fortified with linseed oil.

**Methods:**

Twelve 3 to 4 months old male goats and sheep were randomly allocated into two dietary treatment groups in a 2 (species)×2 (oil levels) factorial experiment. The treatments were: i) goats fed basal diet, ii) goats fed oil-supplemented diet, iii) sheep fed basal diet, and iv) sheep fed oil-supplemented diet. Each treatment group consisted of six animals. Animals in the basal diet group were fed with 30% alfalfa hay and 70% concentrates at a rate equivalent to 4% of their body weight. For the oil treatment group, linseed oil was added at 4% level (w:w) to the concentrate portion of the basal diet. Growth performance of the animals was determined fortnightly. Digestibility study was conducted during the final week of the feeding trial before the animals were slaughtered to obtain rumen fluid for rumen fermentation characteristics study.

**Results:**

Sheep had higher (p<0.01) average daily weight gain (ADG) and better feed conversion ratio (FCR) than goats. Oil supplementation did not affect rumen fermentation in both species and improved ADG by about 29% and FCR by about 18% in both goats and sheep. The above enhancement is consistent with the higher dry matter and energy digestibility (p<0.05), as well as organic matter and neutral detergent fiber digestibility (p<0.01) in animals fed oil- supplemented diet. Sheep had higher total volatile fatty acid production and acetic acid proportion compared to goat.

**Conclusion:**

The findings of this study suggested that sheep performed better than goats when fed a fattening diet and oil supplementation at the inclusion rate of 4% provides a viable option to significantly enhance growth performance and FCR in fattening sheep and goats.

## INTRODUCTION

Asia-Pacific region is home to about half of the global two billion goats and sheep which play a vital socio-economic role in the region [1]. Lately, meat and milk from these two small ruminant species have received increasing attention by both, the producers and researchers, due to the high demand of these products across a number of Asian countries. However, the current traditional low-input farming system of goats and sheep across many Asian countries hinders the growth of the industry to meet the increasing demand. This traditional production system involved animal feeding on poor quality feed with almost no supplementary feeding resulting in animals achieving low growth rate and take longer time to reach market weight [2]. Thus, there is a need to improve the current production system in order to achieve higher productivity. This includes the feeding of concentrates [3] to enhance feed intake, higher weight gain and better feed conversion ratio (FCR) [4]. Fortification of feed with the appropriate dietary fat, such as sunflower, fish and linseed oils, besides increasing the energy density of the diet to improve growth of the animals at similar feed intake [5], also improves the desired fatty acids content in the meat [6], which finally can benefits the consumers. However, some studies seemed to suggest that oil supplementation reduced feed digestibility [7] while others [5,8] found otherwise. The inconsistency could be due to the type of oil used in the different studies, which are made up of different composition of fatty acids. An appropriate level of oil supplementation (especially for unsaturated oil) also plays a role in a sound dietary oil supplementation without interference in feed digestibility or rumen function. Our previous *in vitro* study [9] which used 4% linseed oil supplementation has shown minimal effect on rumen microbial population. Therefore, the same level linseed oil supplementation (4%) was used in this follow-up *in vivo* study.

Although belonging to the same subfamily *Caprinae*, goat and sheep are of two different species; the former, being a browser is highly selective in feeding compared to sheep which is a grazer [10]. Available information on efficiency of feed utilization between goat and sheep has been inconsistent with several studies reported sheep had higher growth performance than goats [11]. In a recent *in vitro* study, Candyrine et al [9] reported that goats had better rumen fermentation characteristics, including higher volatile fatty acids (VFAs) production and higher population of cellulolytic bacteria compared to sheep, suggesting that goats could be more superior in digesting feed materials. Direct co-comparing the *in vivo* efficiency of feed utilization between goat and sheep is scanty. Therefore, this study was conducted with the objective to co-compare the growth performance, nutrient digestibility and rumen fermentation characteristics in goats and sheep fed high concentrate fattening diet with and without linseed oil supplementation.

## MATERIALS AND METHODS

### Animals and feeding

Animals were cared in accordance to the Code of Practice for the Care and Use of Animal for Scientific Purposes Universiti Putra Malaysia. Twelve Boer-crosses male goats (14.28±2.17 kg) and twelve male Dorper lambs (16.56±2.62 kg) of between three to four months old were used in this study. Animals were randomly allocated to a 2 (species)×2 (diet) factorial design experiment of four treatment groups: i) goat fed control diet, ii) goat fed oil-supplemented diet, iii) sheep fed control diet, and iv) sheep fed oil-supplemented diet. Each treatment group consisted of six animals (replicates). The animals were housed in individual pens with free access to clean drinking water and mineral blocks.

Diet for the control group consisted of 30% alfalfa hay and 70% commercial concentrates (FFM Marketing Sdn. Bhd., Selangor, Malaysia). The commercial concentrate was made up of the following ingredients: corn, soybean meal, rice bran, wheat, wheat flour, wheat pollard, molasses, distiller’s dried grains with solubles, palm kernel cake, soya hulls, limestone, salt, dicalcium phosphate, vitamins, trace minerals and feed additives. For the oil supplemented diet, linseed oil (5.24% of C16:0, 3.35% of C18:0, 19.42% of C18:1n-9, 16.06% of C18:2n-6, 0.34% of C18:3n-6, and 55.59% of C18:3n-3 of the total identified fatty acids) was added at 4% to the concentrate (w:w) and thoroughly mixed. Dry matter (DM), ash, and organic matter, crude protein (CP), and ether extract of the feed were determined according to AOAC [12]. Neutral detergent fiber (NDF), acid detergent fiber (ADF), and acid detergent lignin were determined according to Van Soest et al [13]. The chemical compositions of experimental feed are reported in Table 1. Fatty acids content of linseed oil was determined according to method of Folch et al [14] with minor modifications described by Ebrahimi et al [15]. Feed were offered at 4% (DM basis) of body weight (BW) of the animal at equal portion twice a day (0800 and 1700 h) and the refusal was collected daily before the morning feeding. The amount of feed offered was adjusted every fortnight upon weighing of the animals, which was done prior to morning feeding. Fortnight BW were used to determine body weight gain (BWG), average daily gain (ADG), FCR for the 100 days feeding trial.

During the final week of the study, animals were placed in the metabolic crates and digestibility trial was conducted using the total collection technique for seven days. The total feces produced every day were weighed, recorded and 10% of the total daily feces for seven days were pooled for each animal. The pooled feces were dried at 60°C for 48 hours before being subjected to proximate analysis. The apparent nutrient digestibility was calculated according to the formula:

Digestibility (%)=[(nutrient in feed-nutrient in feces)/nutrient in feed]×100

At the end of the feeding trial, animals were slaughtered in accordance with the standard procedures (MS 1500:2004 of the Department of Standards Malaysia, 2004) at a slaughter house certified by the Department of Veterinary Services Malaysia for collection of samples.

Rumen content was collected directly from the rumen of the animals after slaughtering and was strained through four layers of cheesecloth. Approximately 5 mL of the strained rumen fluid was taken for microbial population study while another 10 mL of the fluid was mixed with 2 mL 25% meta-phosphoric acid (w:v) for volatile fatty acid analysis. All samples were kept on ice during sampling and kept in −20°C freezer pending for further analysis.

### Volatile fatty acids analysis

The rumen fluid samples for VFA analysis were centrifuged at 10,000 g for 20 min and the supernatant was analysed for total VFA, acetic, propionic and butyric acids content by using Agilent 7890A gas–liquid chromatography (Agilent Technologies, Palo Alto, CA, USA).

### Microbial population

In the study, the targeted microbes of interest were total bacteria, protozoa, fungi and methanogen. Since supplementation of linseed oil, containing unsaturated fatty acids, was one of the dietary factors in this study, we specifically measured the population of methanobacteriales and *Butyrivibrio fibrisolvens* (*B. fibrisolvens*) because of their potential role in biohydrogenation of unsaturated fatty acids. On the other hand, fiber degrading bacteria, namely, *B. fibrisolvens*, *Fibrobacter succinogenes*, *Ruminococcus albus*, and *Ruminococcus flavefaciens* were studied because it is known that the supplementation of oil (especially unsaturated) is toxic towards this group of bacteria. In addition, the responses of these microbes to dietary treatments between goat and sheep were of interest to this study as they reflect the distinction in digestive capability between the two species, if any.

The samples for microbial quantification study were centrifuged at 10,000 g for 10 min and DNA was extracted from the precipitate by using QIAamp DNA Stool Mini Kit (Qiagen Inc., Valencia, CA, USA) according to manufacturer’s instruction. The samples were subjected to 95°C lysis temperature to account for cells that are difficult to lyse. The extracted DNA was then subjected to quantitative real-time polymerase chain reaction (q-PCR) to quantify the population of the targeted microbes according to the methods previously described by Candyrine et al [9]. Briefly, the q-PCR reaction was made up of 25 μL mixture, consisting of 12.5 μL of SYBR Green Supermix, 1 μL reverse primer (of the particular targeted microbes), 1 μL forward primer (of the particular targeted microbes), 8.5 μL of DNase- free water, and 2 μL of extracted DNA. The primers used for each targeted microbes, as well as the annealing temperature for each primers are shown in Table 2. Real time quantitative PCR was performed for the extracted DNA using the BioRad CFX96 Touch (BioRad, Hercules, CA, USA). Melting curve analysis was done to confirm the specificity of the amplification of primers for each targeted microbes. The results of the microbial quantification study was analysed by absolute quantification against the standard curves which were constructed using serial dilution of plasmid DNA for each targeted microbes.

### Statistical analysis

All results for growth performances, digestibility and rumen fermentation characteristics were analyzed as 2×2 factorial design using general linear model of the SAS statistical software version 9.3 (SAS Institute, Cary, NC, USA) to study the effect of species (goat vs sheep), diet (control vs oil supplementation) as well as interaction between the two factors. Since there was no interaction effect between the main factors for the growth performance, nutrient digestibility and rumen fermentation parameters, and there were only two treatments within each treatment variable, multiple comparisons of means between treatments were not carried out. Probability was considered at p<0.05 and p<0.01.

## RESULTS AND DISCUSSION

### Growth performance

Total BWG for sheep was higher (p<0.01) than that of goat (Table 3) and this had resulted in better FCR in sheep than goat (p<0.01). No interaction effect between species and oil supplementation was observed. The higher BWG and better FCR in sheep compared to goats could be attributed to the higher intake of sheep, which been grazers, are less selective in what were offered to them compared to goats (browsers) under the confined feeding protocol applied in this study. The above results is in agreement with those of the earlier study by Aboud et al [16] that higher DM intake and BWG were recorded in pen-fed sheep than goats but not under grazing condition [17]. Although average feed intake was higher in sheep than goats but when the feed intake was adjusted to per unit BW of the animal (feed intake/BW) no significant difference was observed between species. Result of this study suggests that sheep are better convertors of high quality feed to BWG as compared to goats.

As expected, FCR improved with oil supplementation in both species (p<0.01), because the caloric value of oil (nine calories/g) is 2.25 folds higher than that of carbohydrate (four calories/g) [18] and thus oil fortified diet had higher energy density resulting in higher energy intake at similar feed intake level. Oil supplementation has also been previously reported to increase growth efficiency in lamb [4]. The oil supplementation in the present study has resulted in 43 g (or 30%) and 67 g (or 29%) increase in the ADG in goats and sheep, respectively, as compared to their counterparts without oil supplement. This increase is definitely economically attractive from the producers’ point of view.

### Nutrient intake and apparent digestibility

Nutrient intake, digestibility and digestible nutrient intake data are presented in Table 4. Because of the higher feed intake in sheep and no species differences in nutrient digestibility coefficient (except for CP), digestible nutrient intakes were generally higher in sheep (p<0.05) than goats. This result thus explains for the better FCR and the higher growth rate of sheep as compared to goats (Table 3). Interestingly, oil supplementation did not adversely affect, and in fact in some cases even enhanced nutrient digestibility and thus digestible nutrient intakes of most nutrients. The above, together with the higher feed intake again explained for the higher growth rate in animals, irrespective of species, fed oil fortified diets.

As pointed out earlier, published results which co-compared the efficiency of digestive capacity between goats and sheep are inconsistent with the general agreement that there are no species differences between goats and sheep in apparent digestibility [2] except that goats may perform better under browsing condition [19]. Similarly, while several studies seem to suggest that oil supplementation reduced feed digestibility [7], others [5,8] and the present study found otherwise. The inconsistency could be due to the type of oil used in the different studies, which are made up of different composition of fatty acids. Zhang et al [20] stated that fatty acids with higher degree of unsaturation are more toxic towards the rumen microbes, thus reducing digestibility. Therefore, the improved digestibility in some nutrients with the inclusion of linseed oil observed in this study contradicts the above and could possibility suggest that the rumen microbes has adapted to the 4% linseed oil supplementation in the concentrates. It is also possible that the oil supplementation resulted in reduction of protozoa [21], leading to a higher population of bacteria, hence, increased in feed digestibility [22]. However, the above were not shown in the results of this study. Regardless of the above, our study clearly showed that fattening diet fortified with 4% linseed oil enhanced both nutrient and digestible nutrient intakes which led to significant increased in growth rate and better FCR in the animals.

### Rumen fermentation

Results on rumen fermentation parameters are shown in Table 5 and 6. There were significant differences in molar proportions of acetic and butyric acids between species with significantly higher acetic acid but lower butyric acid in sheep than goat (Table 5). Total VFA production was also higher in sheep (p< 0.05) compared to goats. On the other hand, oil supplementation did not affect VFA production, and no interaction was observed between the two factors.

Total VFA production obtained in this study (51.67 and 56.72 mmol/L, respectively for goats and sheep, means of both diet in each species) was slightly lower than the 67.0 and 63.2 mmol/L, respectively for goats and sheep, reported by Saini et al [23]. While the higher molar proportion of acetic acid in sheep compared to goats seems to suggest that sheep was better in digesting fiber component of the feed, however, this advantage was not observed in this study (Table 4). With the higher total VFA production and molar proportion of acetic acid, it is expected that sheep have higher available energy to support growth and possibly also higher tendency to biosynthesize fats.

Oil supplementation did not affect total VFA production, suggesting that the level of oil supplementation (4% in the concentrates) used for this study did not interfere with the rumen function which thus help to improve BWG and FCR (Table 3) as discussed earlier.

There were no significant differences between species in the population of total bacteria and fiber digesting bacteria (except for *B. fibrisolvens*) (Table 6) and thus is consistent with the non-significant differences in DM and fiber (NDF and ADF) digestibility between goats and sheep (Table 4). Species differences were only observed in the population of *B. fibrisolvens*, total methanogens and methanobacteriales (p<0.01) with sheep having higher population of *B. fibrisolvens*, but lower population of total methanogens and methanobacteriales than goats (p<0.01). *B. fibrisolvens* has been reported to be involved in fibre degradation [24] and could have contributed to some extent in fiber digestion. Thus the higher production of total VFA and acetic acid proportion (Table 5) in sheep compared to goats, could be attributed to the higher population of *B. fibrisolvens* in sheep than goats (6.6 vs 6.17 log10 copy number/mL; p = 0.001). On the other hand, methanogens are responsible in utilizing hydrogen in the process of methanogenesis. The higher population of methanogens in the goats could have diverted the use of available hydrogen for methane formation, which is a loss of energy from the diet [25] and decreased energy efficiency of animals [26] and thus leading to the lower feed efficiency and slower growth in the goats.

Oil supplementation did not alter microbial population, and also no interaction between species and oil supplementation. Although contradicted with the earlier report that fatty acids, particularly those with higher degree of unsaturation (such as linseed oil), are toxic towards the rumen microbes thus reducing digestibility [20], the present finding is in line with the growth, nutrient digestibility and VFA production data in this study. It is possible that feeding of high concentrates diet, as practiced in this study, had resulted in high passage rate of feed through the rumen and thus reduces the exposure time of rumen microbes to the toxic effect of unsaturated fatty acids on rumen microbes

## CONCLUSION

Results of this study showed that sheep fed fattening diet with or without linseed oil supplementation had better BWG, and FCR compared to goats. Supplementation of linseed oil at the level used in this study did not interfere with rumen function. Meanwhile, it improved the BWG by about 67 g/d (or 29% increase) and improvement in FCR by about 18% in sheep; while in goats, 43 g/d (or 30% increase) BWG and 19% improvement in FCR were observed. The above advantage of oil supplementation on growth performance and FCR would be an attractive option for farmers to produce quality meat from goats and sheep to meet the increasing demand for these products in many Asian countries.

## Figures and Tables

**Table 1:** Chemical composition (dry matter basis, n = 3) of the alfalfa hay, commercial concentrate and experimental diets without (control) and with oil supplement (Plus oil)

Chemical composition (%)	Alfalfa hay	Commercial concentrate	Diet	

			Control	Plus oil
Dry matter	83.44	87.36	87.38	87.35
Ash	9.95	8.07	9.30	8.45
Crude protein	17.93	12.94	18.23	17.39
Ether extract	1.30	3.50	3.22	6.12
Neutral detergent fiber	35.46	42.85	37.59	40.97
Acid detergent fiber	22.12	17.77	20.10	20.51
Acid detergent lignin	7.84	4.63	5.56	5.74
Gross energy (MJ/kg)	17.63	17.68	17.88	18.26

**Table 2:** Primers used for quantitative real-time polymerase chain reactions

Targeted microbes	R/F	Sequence 5′ to 3′	Product size (bp)	Reference
Total bacteria	R	CCATTGTAGCACGTGTGTAGCC	145	Koike and Kobayashi [27]
	F	CGGCAACGAGCGCAACCC		
Total protozoa	R	GCTTTCGWTGGTAGTGTATT	223	Sylvester et al [28]
	F	CTTGCCCTCYAATCGTWCT		
Total fungi	R	CAAATTCACAAAGGGTAGGATGATT	121	Lane [29]
	F	GAGGAAGTAAAAGTCGTAACAAGGTTTC		
Total methanogens	R	CGGTCTTGCCCAGCTCTTATTC	160	Zhou et al [30]
	F	CCGGAGATGGAACCTGAGAC		
Methanobacteriales	R	TACCGTCGTCCACTCCTT	343	Yu et al[31]
	F	CGWAGGGAAGCTGTTAAGT		
*Fibrobacter succinogenes*	R	CGCCTGCCCCTGAACTATC	122	Lane [29]
	F	GTTCGGAATTACTGGGCGTAAA		
*Ruminococcus albus*	R	CCTCCTTGCGGTTAGAACA	175	Koike and Kobayashi [27]
	F	CCCTAA AAGCAGTCTTAGTTCG		
*Ruminococcus flavefaciens*	R	CCTTTAAGACAGGAGTTTACAA	259	Koike and Kobayashi [27]
	F	TCTGGAAACGGATGGTA		
*Butyrivibrio fibrisolvens*	R	CCAACACCTAGTATTCATC	417	Boeckaert et al [32]
	F	GYGAAGAAGTATTTCGGTAT		

**Table 3:** Feed intake, body weight gain, average daily gain, and feed conversion ratio of goat and sheep fed with and without linseed oil supplementation over 100 days experiment

Items	Goat		Sheep		SEM	Significance		
		
	Control	Oil	Control	Oil		Sp	Diet	Sp×diet
Intake (kg)	71.90	96.03	92.05	107.57	4.094	0.018	0.841	0.905
Intake/body weight	3.06	3.05	2.84	2.63	0.062	0.011	0.312	0.386
BWG (kg)	10.06	14.37	16.77	23.55	1.430	0.002	0.175	0.554
ADG (g)	100.60	143.67	167.70	235.50	143.007	0.002	0.018	0.555
FCR	7.17	5.78	5.65	4.60	0.269	0.004	0.007	0.668

SEM, standard error of the mean; Sp, species, Sp×diet, species×diet; BWG, body weight gain; ADG, average daily gain; FCR, feed conversion ratio.

**Table 4:** Nutrient intake, apparent nutrient digestibility (%) and digestible nutrient intake of goat and sheep fed high concentrate diet with and without linseed oil supplementation

Items	Goat		Sheep		SEM	Significance		
		
	Control	Oil	Control	Oil		Sp	Diet	Sp×diet
Intake (g/d)								
DM	718.99	960.27	920.55	1,075.72	40.942	0.034	0.110	0.531
OM	654.07	782.94	837.38	984.80	50.638	0.007	0.002	0.634
CP	131.07	148.72	167.81	187.06	9.069	0.006	0.008	0.698
NDF	270.27	350.38	346.02	440.71	24.570	0.007	0.002	0.628
ADF	144.52	175.40	185.02	220.63	11.476	0.007	0.001	0.571
Digestibility (%)								
DM	74.83	75.79	73.52	76.08	0.377	0.441	0.017	0.234
OM	76.63	77.55	75.25	77.91	0.377	0.432	0.014	0.192
CP	85.06	83.97	82.06	83.90	0.384	0.042	0.589	0.052
NDF	60.43	64.40	58.16	65.14	0.931	0.585	0.001	0.288
ADF	60.19	57.31	57.25	62.05	0.773	0.517	0.493	0.014
Energy	73.95	75.61	73.27	75.98	0.422	0.833	0.011	0.495
Digestible nutrient intake (g/d)								
DM	538.02	727.79	676.79	818.40	43.553	0.053	0.009	0.670
OM	501.22	607.17	630.13	767.26	41.164	0.011	0.002	0.542
CP	111.49	124.88	137.70	156.95	7.664	0.011	0.008	0.562
NDF	163.33	225.64	201.24	287.08	18.100	0.020	0.001	0.434
ADF	86.99	100.52	105.92	136.90	7.939	0.012	0.001	0.230
Energy (MJ/kg)	9.58	12.82	11.91	16.57	0.749	0.010	0.002	0.479

SEM, standard error of the mean; Sp, species, Sp×diet, species×diet; DM, dry matter; OM, organic matter; CP, crude protein; NDF, neutral detergent fiber; ADF, acid detergent fiber.

**Table 5:** Volatile fatty acids content in goat and sheep under different dietary treatments

Items	Goat		Sheep		SEM	Significance		
		
	Control	Oil	Control	Oil		Sp	Diet	Sp×diet
VFA (mol/100 mol)								
Acetic	39.77	38.87	46.62	46.48	1.211	0.003	0.785	0.843
Propionic	25.10	22.08	24.30	24.91	1.007	0.669	0.611	0.447
Butyric	20.14	19.74	16.37	14.79	0.703	0.001	0.284	0.520
Others	14.99	19.32	12.72	13.83	0.969	0.060	0.169	0.402
Acetic:propionic	1.64	1.80	1.99	1.92	0.100	0.311	0.845	0.614
Total VFA (mM/L)	54.44	48.89	55.10	58.34	1.177	0.035	0.601	0.061

SEM, standard error of the mean; Sp, species, Sp×diet, species×diet; VFA, volatile fatty acid.

**Table 6:** Rumen microbial population (log_10_ copy number/mL) in goat and sheep under different dietary treatments

Items	Goat		Sheep		SEM	Significance		
		
	Control	Oil	Control	Oil		Sp	Diet	Sp×diet
Total bacteria	10.40	10.00	9.91	10.12	0.162	0.607	0.786	0.389
*Fibrobacter succinogenes*	8.09	8.00	7.62	7.50	0.184	0.228	0.796	0.974
*Ruminococcus albus*	8.74	8.61	9.12	8.94	0.115	0.159	0.537	0.911
*Ruminococcus flavefaciens*	6.50	6.65	6.64	6.78	0.230	0.792	0.779	0.985
*Butyrivibrio fibrisolvens*	6.09	6.25	6.55	6.65	0.071	0.001	0.238	0.831
Total methanogen	7.70	7.69	6.93	6.88	0.105	<0.0001	0.842	0.884
Methanobacteriales	5.79	5.92	5.08	4.96	0.132	0.001	0.966	0.556
Total protozoa	6.95	6.96	6.32	6.61	0.208	0.284	0.739	0.757

SEM, standard error of the mean; Sp, species, Sp×diet, species×diet.
